# Performance evaluation and comparative analysis of different machine learning algorithms in predicting postnatal care utilization: Evidence from the ethiopian demographic and health survey 2016

**DOI:** 10.1371/journal.pdig.0000707

**Published:** 2025-01-09

**Authors:** Daniel Niguse Mamo, Agmasie Damtew Walle, Eden Ketema Woldekidan, Jibril Bashir Adem, Yosef Haile Gebremariam, Meron Asmamaw Alemayehu, Ermias Bekele Enyew, Shimels Derso Kebede

**Affiliations:** 1 Department of Health Informatics, School of Public Health, College of Medicine and Health Sciences, Arbaminch University, Arbaminch, Ethiopia; 2 Department of Health Informatics, School of Public Health, Asrat Woldeyes Health Science Campus, Debre Berhan University, Debre Birhan, Ethiopia; 3 Department of Public Health, Arsi University, Asella, Ethiopia; 4 Department of Public Health, School of Public Health, College of Medicine and Health Sciences, Arbaminch University, Arbaminch, Ethiopia; 5 Department of Epidemiology and Biostatistics, Institute of Public Health, College of Medicine and Health Sciences, University of Gondar, Gondar, Ethiopia; 6 Department of Health Informatics, School of Public Health, College of Medicine and Health Sciences, Wollo University, Dessie, Ethiopia; The University of Sheffield, UNITED KINGDOM OF GREAT BRITAIN AND NORTHERN IRELAND

## Abstract

Postnatal care refers to the support provided to mothers and their newborns immediately after childbirth and during the first six weeks of life, a period when most maternal and neonatal deaths occur. In the 30 countries studied, nearly 40 percent of women did not receive a postpartum care check-up. This research aims to evaluate and compare the effectiveness of machine learning algorithms in predicting postnatal care utilization in Ethiopia and to identify the key factors involved. The study employs machine learning techniques to analyse secondary data from the 2016 Ethiopian Demographic and Health Survey. It aims to predict postnatal care utilization and identify key predictors via Python software, applying fifteen machine-learning algorithms to a sample of 7,193 women. Feature importance techniques were used to select the top predictors. The models’ effectiveness was evaluated using sensitivity, specificity, F1 score, precision, accuracy, and area under the curve. Among the four experiments, tenfold cross-validation with balancing using Synthetic Minority Over-sampling Technique was outperformed. From fifteen models, the MLP Classifier (f1 score = 0.9548, AUC = 0.99), Random Forest Classifier (f1 score = 0.9543, AUC = 0.98), and Bagging Classifier (f1 score = 0.9498, AUC = 0.98) performed excellently, with a strong ability to differentiate between classes. The Region, residence, maternal education, religion, wealth index, health insurance status, and place of delivery are identified as contributing factors that predict postnatal care utilization. This study assessed machine learning models for forecasting postnatal care usage. Ten-fold cross-validation with Synthetic Minority Oversampling Technique produced the best results, emphasizing the significance of addressing class imbalance in healthcare datasets. This approach enhances the accuracy and dependability of predictive models. Key findings reveal regional and socioeconomic factors influencing PNC utilization, which can guide targeted initiatives to improve postnatal care utilization and ultimately enhance maternal and child health.

## Author summary

The study indicated that, among the four experimental designs, tenfold cross-validation using the Synthetic Minority Oversampling Technique (SMOTE) produced the best results. The MLP Classifier, Random Forest Classifier, and Bagging Classifier all performed well in predicting postnatal care (PNC) utilization. The region, residency, maternal education, religion, wealth index, health insurance status, and place of delivery were all significant predictors. The study emphasizes the necessity of correcting class imbalance in healthcare datasets to improve predictive model accuracy and reliability, as well as the impact of geographical and socioeconomic factors on PNC utilization.

## Introduction

According to the World Health Organization (WHO), postnatal care is defined as the care provided to mothers and their newborns immediately after childbirth and for the first six weeks of life. During this period,the majority of maternal and neonatal deaths occur [1]. A large proportion of maternal and neonatal deaths occur during the 48 hours following childbirth and almost all (99%) newborn and maternal deaths happen in developing countries [2,3]. Birth is the period with the greatest risk, during which over 40% of maternal deaths (approximately 290,000 in total) and 5.5 million stillbirths or neonatal deaths occur annually [4].

In developing countries, such as those in Sub-Saharan Africa, the majority of maternal and neonatal deaths occur. It is estimated that 289,000 women die from pregnancy-related complications every year [5]. Every year, 1,208,000 babies die before one month of age is reached. On average, more than 13,000 of these deaths occur each day, accounting for half of all maternal and child mortality worldwide. Additionally, approximately880,000 new-borns are stillborn in Sub-Saharan Africa, a fact often overlooked in policy discussions [4,6]. Figures were worst for countries such as Ethiopia [7], where 90% of women did not receive any postpartum care, followed by Bangladesh (73%) [8], Nepal (72%) [9], and Rwanda (71%) [10]. Other countries presented substantial proportions of women who did not receive any postpartum care, including Cambodia (46%) [11], Kenya (46%) [12], Mali (49%) [13], and Nigeria (46.5%) [14]. On average, in the 30 countries examined, nearly 40% of women did not receive a postpartum care check-up [15].

Inadequate postnatal care in Africa has serious effects. This includes increased maternal mortality from hemorrhage and sepsis. It also interferes with family planning efforts, increases the frequency of pregnancies, and has an impact on breastfeeding and HIV prevention follow-up. Additionally, it contributes to increased infant mortality, infections, and long-term impairments [16–18]. Ethiopia has inadequate postnatal care coverage. Only 17% of women reported receiving a postnatal check-up during the first two days after birth. This is alarming because a major percentage of maternal and newborn mortality occurs during this period [7]. The Sustainable Development Goals (SDGs), specifically SDG-3, seek to reduce newborn and maternal death rates by 2030. The Ethiopian government subsequently approved the Health Sector Transformation Plan (HSTP), which aims to reduce maternal and newborn mortality rates [19].

Unfortunately, postnatal care is not utilized enough in many African countries, including Ethiopia. Therefore, it is crucial to recognize and manage issues that arise during delivery, as well as provide mothers with valuable self-care and newborn health education. Complications and health difficulties can arise without proper postnatal care, which may have long-term implications [20,21]. Sociodemographic and economic factors, accessibility and availability of healthcare services, cultural beliefs, and women’s autonomy all play important roles in determining whether women seek and receive postnatal care [22]. To improve the quality of care and develop appropriate interventions, it is crucial to understand these factors. Additionally, knowledge about the benefits and importance of postnatal care services is a significant factor influencing utilization [23,24]. Postnatal care is a vital strategy for preventing physical and mental impairments among women who have delivered and reducing maternal and child mortality rates [25].

Many studies have harnessed traditional statistical analysis to explore the underlying factors influencing postnatal care utilization [26–29]. Machine learning is a scientific method for developing prediction models. Recent studies have shown that machine learning, including deep learning, can greatly increase prediction performance [30–32]. Machine learning algorithms are rapidly being utilized in public health for disease diagnosis, epidemic surveillance, resource allocation, medication discovery, and remote healthcare delivery. it having enormous potential to improve healthcare, particularly for populations who are less fortunate, by detecting high-risk infants, managing complex interactions, picking relevant variables, improving predictions, and encouraging continuous learning [33,34]. The purpose of this study was to determine the performance of machine learning evaluation and comparison analysis in predicting postnatal care utilization among reproductive-aged women who achieved PNC within two months in Ethiopia. This analysis used secondary data from the Ethiopian Demographic Health Survey to assist future actions aimed at increasing postnatal care utilization.

## Method

### Data sources and population

A cross-sectional study design with a secondary analysis of 2016 DHS data from Ethiopia was used. The 2016 survey was EDHS, which was performed by the Central Statistical Agency (CSA), Federal Minister of Health (FMOH), and Ethiopian Public Health Institute (EPHI), with technical assistance from the International Classification of Functioning [35]. A nationally representative survey of 15,683 women aged 15–49 years was conducted across 645 enumeration areas from January 18—June 27, 2016, via a multistage stratified sample technique. The study focused on looking into the use of postnatal care (PNC) among women of reproductive age, defined as those who had given birth within the five years preceding the survey. The data on PNC utilization, which refers to women’s health check-ups within the first 6 weeks after delivery, were gathered from verbal reports supplied by mothers. The study used a total weighted sample of 7,193 women to ensure that the findings were representative and could be applied to a larger population of reproductive-age women.

### Study features

The study’s outcome variable was postnatal care utilization. PNC utilization is determined by whether a woman had at least one visit or care within the first week after being discharged from a health facility or after having given birth at home. The variable was labeled **’Yes’** if the PNC was received at a health facility and **’No’** otherwise. In this study, sociodemographic characteristics including age, educational level, occupation, religion, marital status, residence, region, and wealth index as well as maternal reproductive health-related factors, such as place of delivery, number of antenatal care (ANC) visits, personal mobile phone, wanted child, age at first sex, and birth order, are utilized as independent variables.

### Data preprocessing

Data preprocessing is an important element in machine learning model development since it includes converting raw data into a format that improves the model’s prediction performance. It consists of several critical phases, including data cleaning, feature engineering, dimensionality reduction, and data splitting, each of which serves a specific purpose in refining the data before analysis.

### Data cleaning

Data cleaning is an important stage in data preprocessing, as it focuses on increasing dataset quality by resolving issues such as outliers, missing values, and class imbalances. Various strategies can be used to address these challenges effectively. To address missing values approaches such as k-nearest-neighbors imputation can be used. This technique fills in missing values by examining the values of the K-nearest neighbors while taking into account the similarity between instances [36–38].

### Class imbalance

A significant difficulty in machine learning is class imbalance, which occurs when one class is underrepresented in comparison to others. Techniques such as the Synthetic Minority Oversampling Technique (SMOTE) can help alleviate this issue by creating synthetic instances of the minority class, thereby balancing class representations in the dataset. This enables the model to make better predictions for rare or minority-class instances. Applying these class imbalance strategies can improve the dataset, ensuring that the model receives high-quality data for training and producing accurate predictions [39–41].

### Feature engineering

Feature engineering is a vital step in data preprocessing, where raw data are transformed into meaningful features that effectively represent the problem being addressed [42,43]. Several techniques are commonly used in this process. One-hot encoding is a common technique used in data preprocessing to convert categorical data into a numerical format. It involves creating binary columns for each category, where the presence of a category is represented as 1 and the absence of a category is represented as 0. One-hot encoding ensures that the models can effectively process and learn from the data by converting categorical variables into a binary representation [44]. Label encoding is another technique used for converting categorical labels into numeric values. It assigns a unique numerical code to each category. This technique is often employed when the ordinal relationship between categories is significant [45]. By performing feature engineering, the data are transformed into a format that can be effectively utilized by machine learning models, enabling them to make accurate predictions and uncover meaningful insights.

### Dimensionality reduction

Dimensionality reduction lowers the number of input variables while retaining the most important features [46]. **Feature selection** is a regularly used strategy for dimensionality reduction. Feature selection strategies discover and maintain characteristics with the greatest predictive power. These methods evaluate the significance of each feature and choose the subset that contributes the most to the model’s performance [42,47]. In addition, the objective of feature selection is to rank and prioritize the most influential predictors within the dataset. This ranking is achieved by calculating the information gain values for each selected variable. To identify the key factors contributing to postnatal care utilization pregnancy, we employed various methods of features of selection, including bortua feature selection method, Select K Best, Chi Square test and a random forest feature importance. Higher information gain values signify that certain variables have strong associations with their respective classes. The top ten information gain values were selected randomly. This method is quite effective for simplifying model complexity and speeding up the processing of machine learning algorithms.The Boruta method is a powerful feature selection strategy that uses a random forest classifier to determine the significance of features. Unlike prior methods, Boruta compares each feature’s importance against randomly produced shadow features, resulting in a more unbiased and efficient feature selection procedure. This contributes to the discovery of truly informative traits while reducing the risk of overfitting or picking irrelevant features [48]. By applying dimensionality reduction techniques like feature selection, we can eliminate redundant or less informative variables, simplifying the model [49] and improving its effectiveness in making accurate predictions on new data.

### Data split

In machine learning approaches, the dataset is typically divided randomly into two parts: a training dataset and a test dataset. The training dataset is used to train the model, whereas the test dataset is used to evaluate the model’s performance by comparing the predicted outcomes to the actual outcomes. In addition to training and test datasets, a validation dataset is often used to fine-tune the model’s parameters. This dataset helps in optimizing the model’s performance by incorporating parameter estimates [50–52]. In the specific study mentioned, the dataset was divided into ten folds using stratified tenfold cross-validation. Stratified tenfold cross-validation is a method in machine learning to assess a model’s performance. It ensures that each data fold maintains the same class distribution as the original dataset, addressing issues related to class imbalance and ensuring that the model’s performance is representative across different classes [53,54].

### Model selection

Once the dataset was split into training and testing sets, we proceeded to choose appropriate machine learning models for predicting postnatal care categories as a binary classification problem. The dataset was divided into two exclusive categories related to postnatal care [45]. We applied fifteen machine learning algorithms for the analysis, including AdaBoost Classifier [55], MLPClassifier [56], SGDClassifier [57], Passive Aggressive Classifier [57], Ridge ClassifierCV[57], Bagging Classifier [57], eXtreme Gradient Boosting (XGBoost) [58,59], Logistic Regression (LR) [60], Gaussian Naive Bayes (GNB) [61,62], Random Forest (RF) [63,64], K-Nearest Neighbors (KNN) [65], Support Vector Machine (SVM) [66], BernoulliNB and Decision Tree (DT) [67] classifiers. Each algorithm was trained on the training set and then evaluated on the testing set to determine its effectiveness in distinguishing between the two categories of postnatal care.

### Performance evaluation of the predictive model

The effectiveness of prediction models was assessed via various measures such as precision, sensitivity, specificity, the F1-score, and the area under the receiver-operating characteristic (AUC-ROC) curve. Precision measures the model’s accuracy in identifying positive cases by calculating the ratio of correctly predicted positives to total predicted positives. Sensitivity, also known as the recall or true positive rate, evaluates the model’s ability to identify all positive cases by calculating the ratio of correctly predicted positives to total actual positives. Specificity evaluates the model’s capability to identify all negative cases by calculating the ratio of correctly predicted negatives to total actual negatives. The F1-score provides a balanced assessment of the model’s accuracy by considering both precision and sensitivity; it represents the harmonic mean of these measures. The AUC-ROC demonstrates the overall model performance by comparing the true positive rate with the false positive rate across different classification thresholds, with a higher value indicating better differentiation between positive and negative classes. These measures were used to evaluate and compare the predictive accuracy of the models in correctly classifying instances [68–70].

According to the confusion matrix above (Table 1), the following lists of recall (sensitivity), (specificity), precision, and accuracy values were derived

$$Recall\;(Sensitivity)=\frac{TP}{TP+FN}$$ (1)

$$Specificity=\frac{TN}{TN+FP}$$ (2)

$$Precision=\frac{TP}{TP+FP}$$ (3)

$$Accuracy=\frac{TP+TN}{TN+TP+FP+FN}$$ (4)

$$F1=2*\frac{Recall*Precision}{Recall+Precision}$$ (5)

**Table 1:** Confusion matrix and different derived metrics adapted from [71].

	Predictive positive		Predictive negative
Actual positive	True Positive (TP)	False Negative (FN)	
Actual negative	False Positive (FP)	True Negative (TN)	

**Fig 1:**
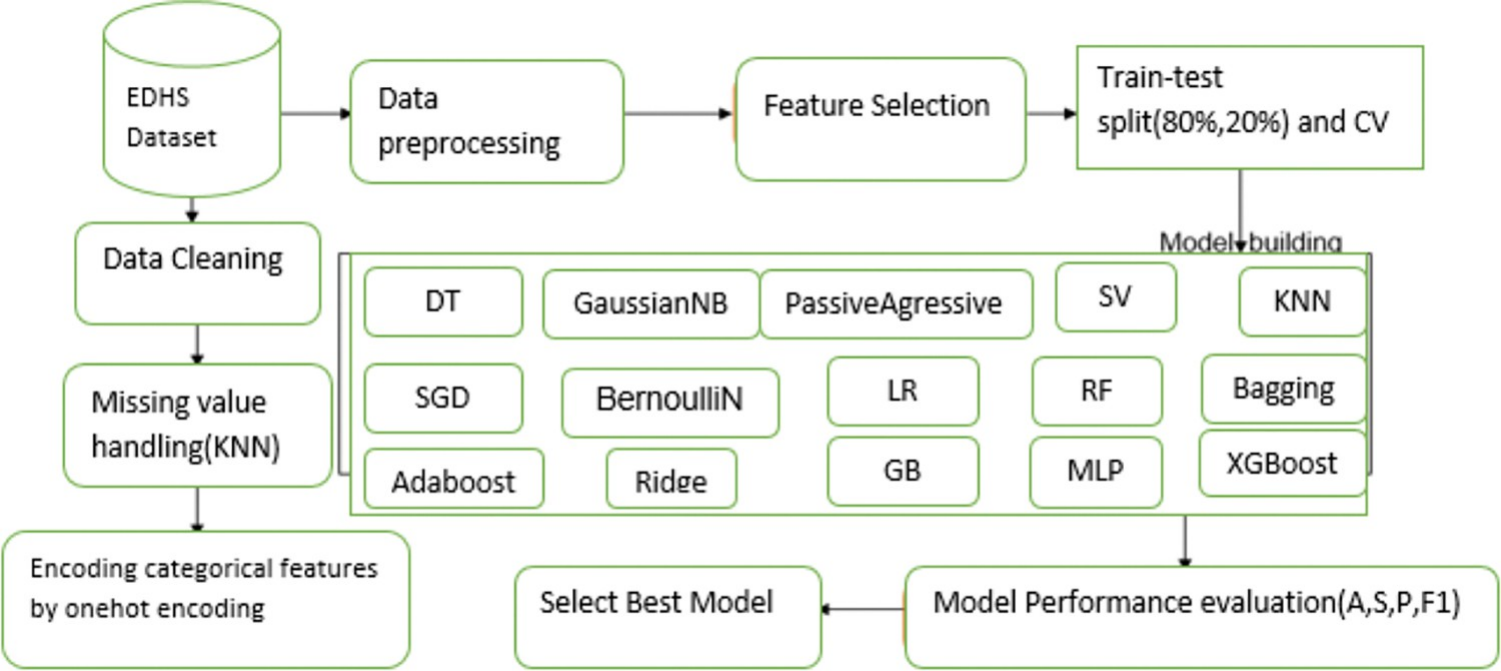
Workflow of machine learning for postnatal care utilization prediction.

The workflow details for the current study are presented (see Fig 1).

### Ethics approval and consent to participate

The EDHS data was used, publicly available on the Demographic and Health Surveys (DHS) website (https://dhsprogram.com/data/available-datasets.cfm), with the committee’s full permission and agreement. We strictly followed all DHS approval rules and regulations. The study’s approach adheres to relevant criteria and can be obtained upon reasonable request after creating an account.

## Results

From the distribution of features with label frequency and percentage in the postnatal care utilization datasets, the majority of women (78.98%) lived in rural areas, with the remainder living in urban settings. The predominant age group for mothers was 20–34 years (70.08%), and a significant portion had no education (60.6%). Most women were married (92.62%), and the majority of pregnancies were wanted at the time (79.81%), yet 61.1% of deliveries occurred at home. Half of the women were classified as poor (50.15%), and most did not own a mobile phone (73.85%). While most husbands were employed (82.14%), only 3.57% of the families had health insurance. Employment among women varied, with 56.68% not working and 43.32% employed. Birth order also varied, with 48.74% having six or more children. In terms of religious affiliation 46.21% were Muslim, and 32.93% were Orthodox. The decision-making process in marriages was often influenced by parents (54.97%). The age at first sex was mostly between 15and19 years (62.59%), and postnatal care utilization was notably low, at only 6.9% (**Table 2**).

**Table 2:** Distribution of features with label frequency and percentage in the PNC care utilization datasets (n = 7193).

Features	Category	Frequency	Percentage
Maternal Age			
	15–19	358	4.98
	20–34	5041	70.08
	35–49	1794	24.94
Region			
	Oromia	1031	14.33
	SNNPR	893	12.41
	Somali	806	11.21
	Tigray	772	10.73
	Amhara	764	10.62
	Afar	647	8.99
	Benishangul	576	8.01
	Gambela	534	7.42
	Harari	411	5.71
	Dire Dawa	384	5.34
	Addis Abeba	375	5.21
Residence			
	Rural	5681	78.98
	Urban	1512	21.02
Maternal Education			
	No education	4359	60.6
	Primary	1942	27
	Secondary	577	8.02
	Higher	315	4.38
Religion			
	Muslim	3324	46.21
	Orthodox	2369	32.93
	Protestant	1338	18.6
	Other	162	2.25
Marital Status			
	Married	6662	92.62
	Divorced	367	5.1
	Widowed	106	1.47
	Single	58	0.81
Owns Mobile Phone			
	No	5312	73.85
	Yes	1881	26.15
Wealth index			
	Poor	3607	50.15
	Rich	2558	35.56
	Middle	1028	14.29
Birth Order			
	First born	1470	20.44
	Second and Third	2217	30.82
	Fourth and more	3506	48.74
Child Wanted			
	Wanted then	5741	79.81
	Wanted later	991	13.78
	Wanted no more	461	6.41
Place of delivery			
	Home	4395	61.1
	Health institution	2798	38.9
Age First Sex			
	8–14	1431	19.89
	15–19	4502	62.59
	20–24	1065	14.81
	25 and above	195	2.71
Husband Education			
	No education	3188	44.32
	Primary	2160	30.03
	Secondary	745	10.36
	Higher	569	7.91
Maternal Work			
	Not working	4077	56.68
	Working	3116	43.32
Decision on your marriage			
	Parents	3954	54.97
	Myself	3012	41.87
	relatives	108	1.5
	Other	61	0.85
Husband Occupation			
	Employed	5908	82.14
	Not Employed	675	9.38
	do not know	79	1.1
Health Insurance			
	No	6936	96.43
	Yes	257	3.57

### Postnatal care utilization

**Fig 2:**
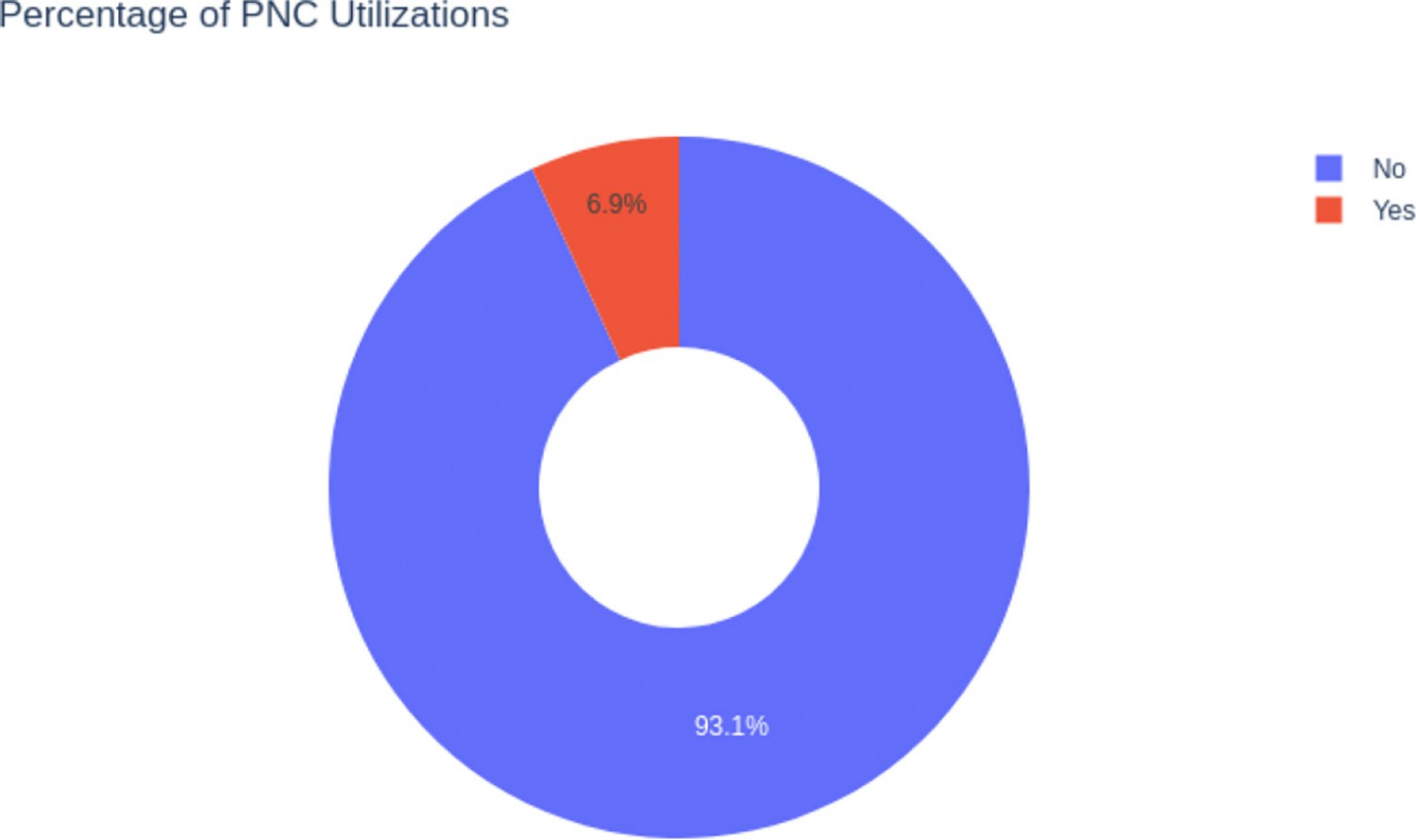
Percentage of PNC Utilizations.

The data show a substantial gap in the use of postnatal care, with only 6.9% of women receiving these critical services, and 93.1% not receiving Postnatal care (**Fig 2**).

### Data preprocessing results

**Fig 3:**
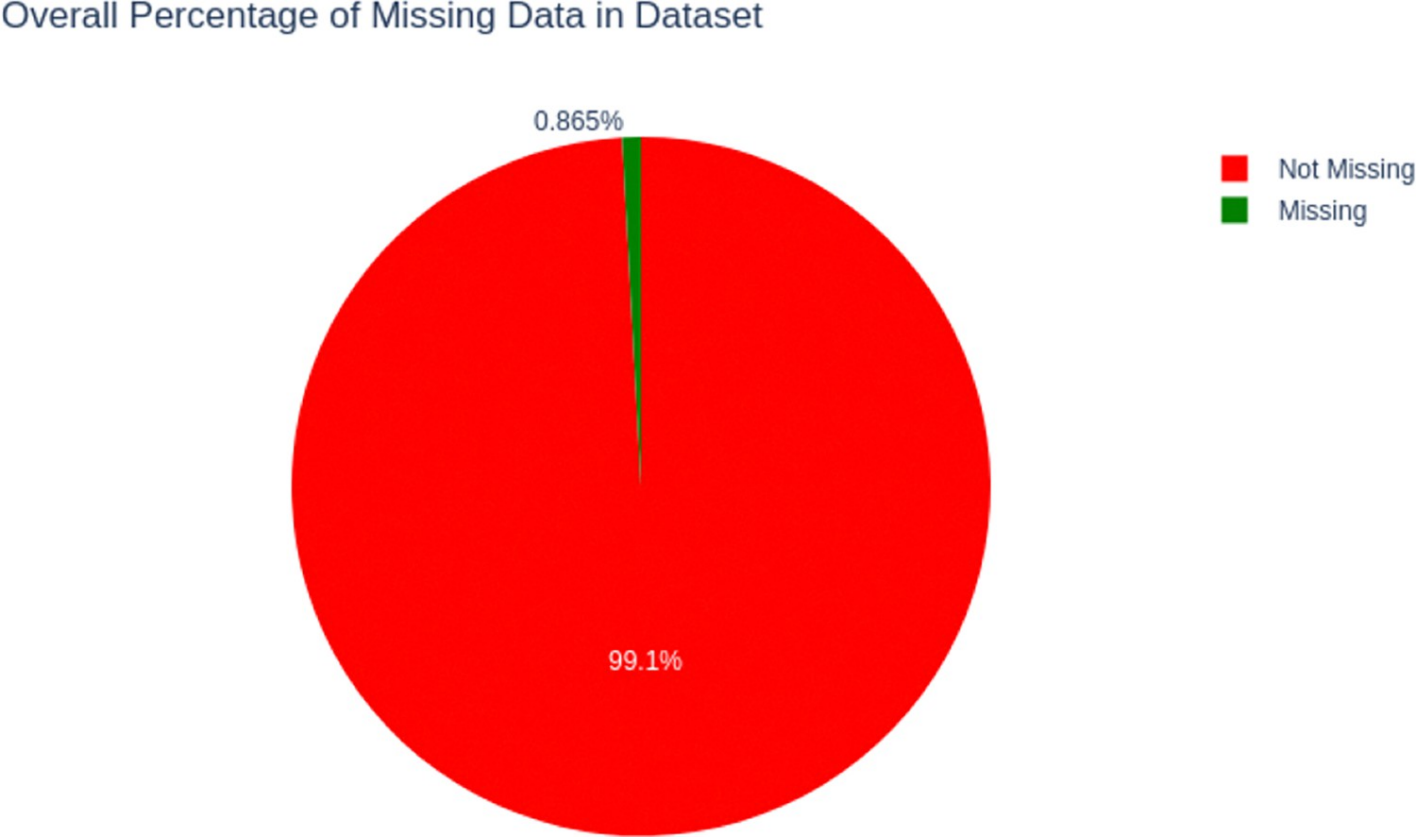
Percentage of Missing Data from PNC Dataset.

The original raw data included incomplete entries, which were addressed via imputation techniques. **Fig 3** depicts the percentage of missing values for each variable, with Husband Education (7.38%), Husband Occupation (7.38%), and Decision on Marriage (0.81%) having the greatest percentages of missing data. The missing values were imputed using the KNN imputation approach. To suit the needs of machine learning models that demand numerical input and output features, we converted the dataset’s categorical variables using one-hot encoding. This resulted in a dataset of eighteen features, including one target feature. One-hot encoding is particularly useful for classification problems since it converts each categorical value into a new column with binary labels (1 or 0).

### ML Analysis of PNC Utilization

The performance of fifteen machine learning algorithms was assessed for the prediction of postnatal care utilization using 18 features discussed in the methods and materials section. A total of 7,193 samples were considered, with 496 utilizing postnatal care and 6,697 samples without utilization. In this study, we evaluated the performance of various machine learning algorithms for predicting postnatal care utilization using four experimental methods: Train-test split (80%-20%) without balancing the training dataset by SMOTE, Train-test split (80%-20%) balancing the training dataset by SMOTE, stratified 10-fold cross-validation without balancing the training dataset by SMOTE, and Stratified 10-fold cross-validation balancing the training dataset by SMOTE. Performance was measured using accuracy, precision, recall, F1-score, and AUC-ROC metrics.

### Experiment 1: Train-test split (80%-20%) before balancing by SMOTE

**Fig 4:**
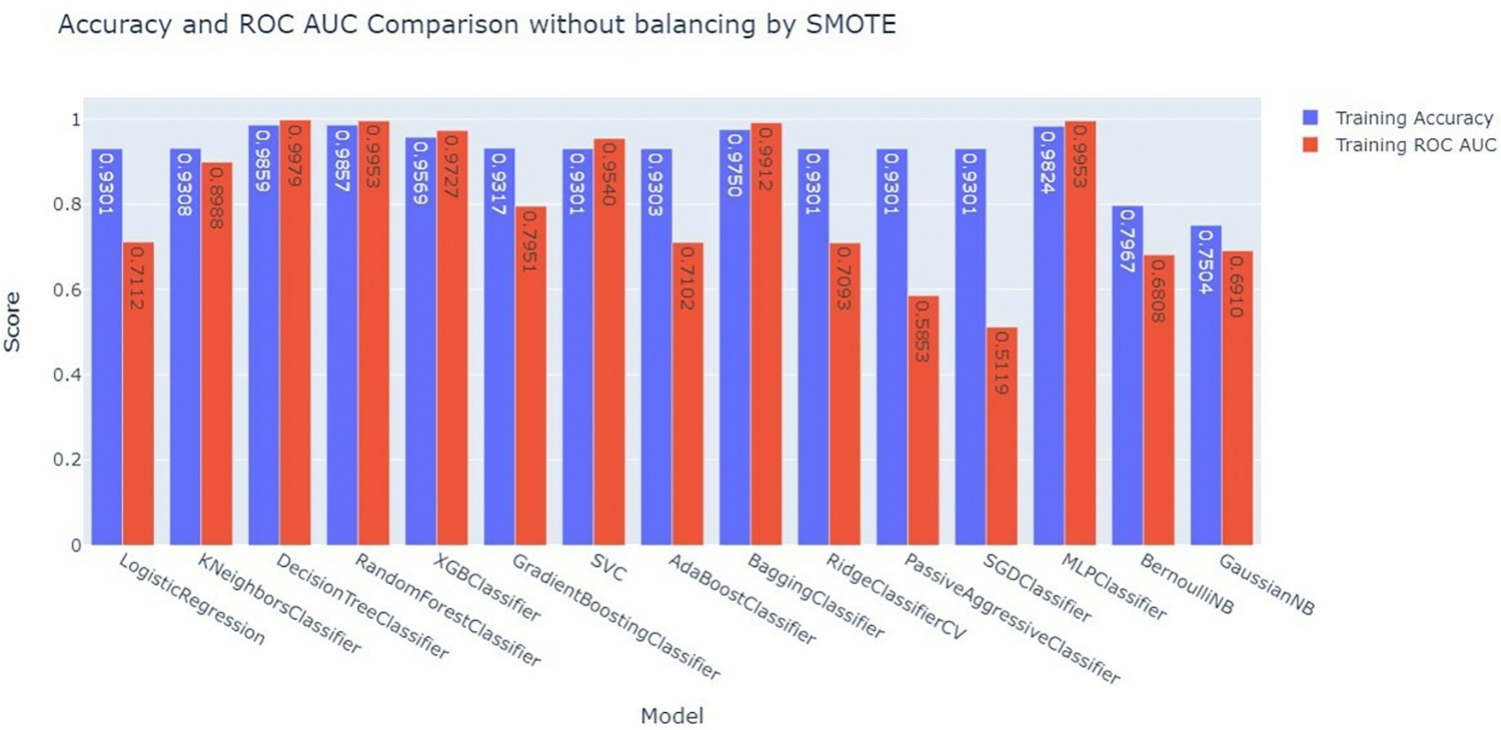
Training accuracy and ROC-AUC Comparison before balancing by SMOTE.

To predict PNC utilization, different machine learning models have been applied to postnatal care utilization data. Before SMOTE was used, some models achieved high training accuracy, but the high accuracy and ROC AUC values suggest potential overfitting. Despite the excellent accuracy of most models, ROC AUC ratings demonstrated considerable disparities in their ability to differentiate across classes. Logistic Regression, while generating a moderate accuracy of approximately 93%, displayed a lower ROC AUC of approximately 0.71, indicating that its ability to correctly differentiate between positive and negative outcomes was relatively weaker than that of models that are more complex. The comparison also revealed the underperformance of simpler models, such as BernoulliNB and GaussianNB, which had the lowest accuracy and ROC AUC scores, with accuracies ranging between 70% and79% and ROC AUCs of approximately 0.69. These models struggled to handle the dataset’s uneven nature, resulting in low classification performance (**Fig 4**).

The performance metrics table provides a detailed comparison of various models used to predict postnatal care utilization, showing significant differences in prediction ability. The measures evaluated include test accuracy, sensitivity, specificity, precision, and the F1 score, each of which provides a unique perspective on model performance under various circumstances. While test accuracy is relatively good across most models, ranging from 0.7526–0.9347, it does not fully represent model performance, as indicated by poor sensitivity ratings in most cases. sensitivity, which measures a model’s ability to correctly detect true positives, is particularly low in models like Logistic Regression, Random Forest, and Gradient Boosting, where it falls to zero. The models appear to have failed to identify any positive examples, making their predictions useful for identifying a vital minority class: postnatal care usage. Despite having a lower test accuracy (0.7526), the GaussianNB model has the highest sensitivity (0.5000), showing that it is more effective than other models at finding positive cases, despite poorer precision and F1-scores. The results reveal a fundamental issue with predicting postnatal care utilization; most models struggle to detect positive cases, as shown by their poor sensitivity and F1 scores. This emphasizes the significance of optimizing models or implementing measures such as rebalancing the dataset, modifying model parameters, or investigating alternative models better suited to addressing class imbalance (**Table 3**).

**Table 3:** Model comparison using multiple metrics before balancing by SMOTE(Train-Test Split (80%-20%)).

Model	Test Accuracy	Sensitivity	Specificity	Precision	F1-score
LogisticRegression	0.9347	0.0000	1.0000	0.0000	0.0000
KNeighborsClassifier	0.9319	0.0426	0.9941	0.3333	0.0755
DecisionTreeClassifier	0.8770	0.1489	0.9279	0.1261	0.1366
RandomForestClassifier	0.9215	0.0000	0.9859	0.0000	0.0000
XGBClassifier	0.9249	0.0213	0.9881	0.1111	0.0357
GradientBoostingClassifier	0.9326	0.0000	0.9978	0.0000	0.0000
SVC	0.9347	0.0000	1.0000	0.0000	0.0000
AdaBoostClassifier	0.9347	0.0000	1.0000	0.0000	0.0000
BaggingClassifier	0.9187	0.0426	0.9799	0.1290	0.0640
RidgeClassifierCV	0.9347	0.0000	1.0000	0.0000	0.0000
PassiveAggressiveClassifier	0.8999	0.0426	0.9599	0.0690	0.0526
SGDClassifier	0.9347	0.0000	1.0000	0.0000	0.0000
MLPClassifier	0.8958	0.0745	0.9532	0.1000	0.0854
BernoulliNB	0.7992	0.3936	0.8275	0.1375	0.2039
GaussianNB	0.7526	0.5000	0.7703	0.1320	0.2089

**Fig 5:**
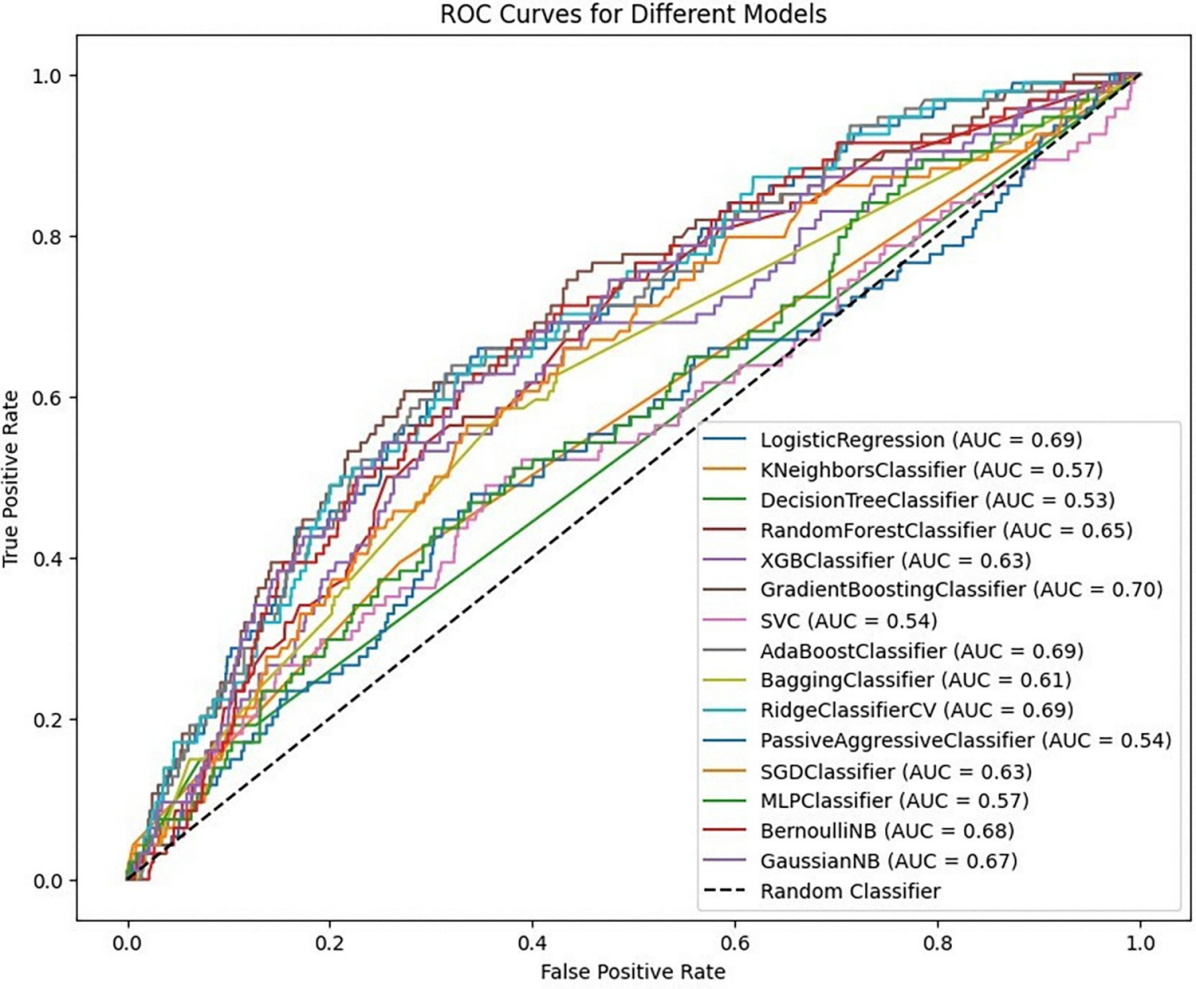
ROC-AUC comparison before balancing by SMOTE (80%, 20%).

Multiple machine learning models have been used to evaluate the usage of postnatal care data. The ROC curves shown in **Fig 5** provide a detailed evaluation of various machine learning models for predicting postnatal care utilization. The area under the curve (AUC) is an important metric since it measures each model’s ability to differentiate between positive and negative classes at all threshold levels. The AUC values differ dramatically among the models, indicating different levels of prediction ability. The GradientBoostingClassifier has the highest AUC of 0.70, indicating that it is the best model for discriminating between classes in this dataset. The LogisticRegression, AdaBoostClassifier, and RidgeClassifierCV models all perform relatively well, with AUC values of 0.69. Several models, including SGDClassifier, KNeighborsClassifier, ExtraTreesClassifier, LPMClassifier, and DecisionTreeClassifier, perform poorly, with AUCs ranging from 0.50 to 0.57, showing that they are just marginally better than random guessing (**Fig 5**).

### Experiment 2: Train-test Split (80%-20%) after balancing by SMOTE

**Fig 6:**
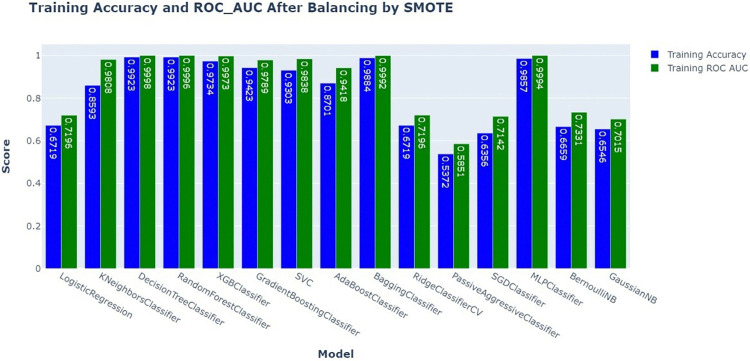
Training accuracy and ROC-AUC comparison after balancing by SMOTE.

The bar chart compares the training accuracy and ROC AUC scores of various models after applying SMOTE to balance the dataset. The blue bars represent training accuracy, whereas the green bars represent training ROC AUC scores. After applying SMOTE, most models achieve high training accuracy, with values close to or above 0.9. However, the ROC AUC scores vary more across models, with Decision Tree Classifier (AUC = 0.9998), Random Forest Classifier (AUC = 0.9996), MLP Classifier (AUC = 0.9994), Bagging Classifier (AUC = 0.992), and XGBoost Classifier (AUC = 0.9973) showed that the highest ROC-AUC scores. The values of the passive aggressive classifier and GaussianNB, which initially had lower ROC-AUC values after being balanced by SMOTE, were approximately 0 0.6147 and 0.7015, respectively. The application of SMOTE has enhanced the performance of most models, making them more reliable for predicting postnatal care (PNC) utilization (**Fig 6**).

**Fig 7:**
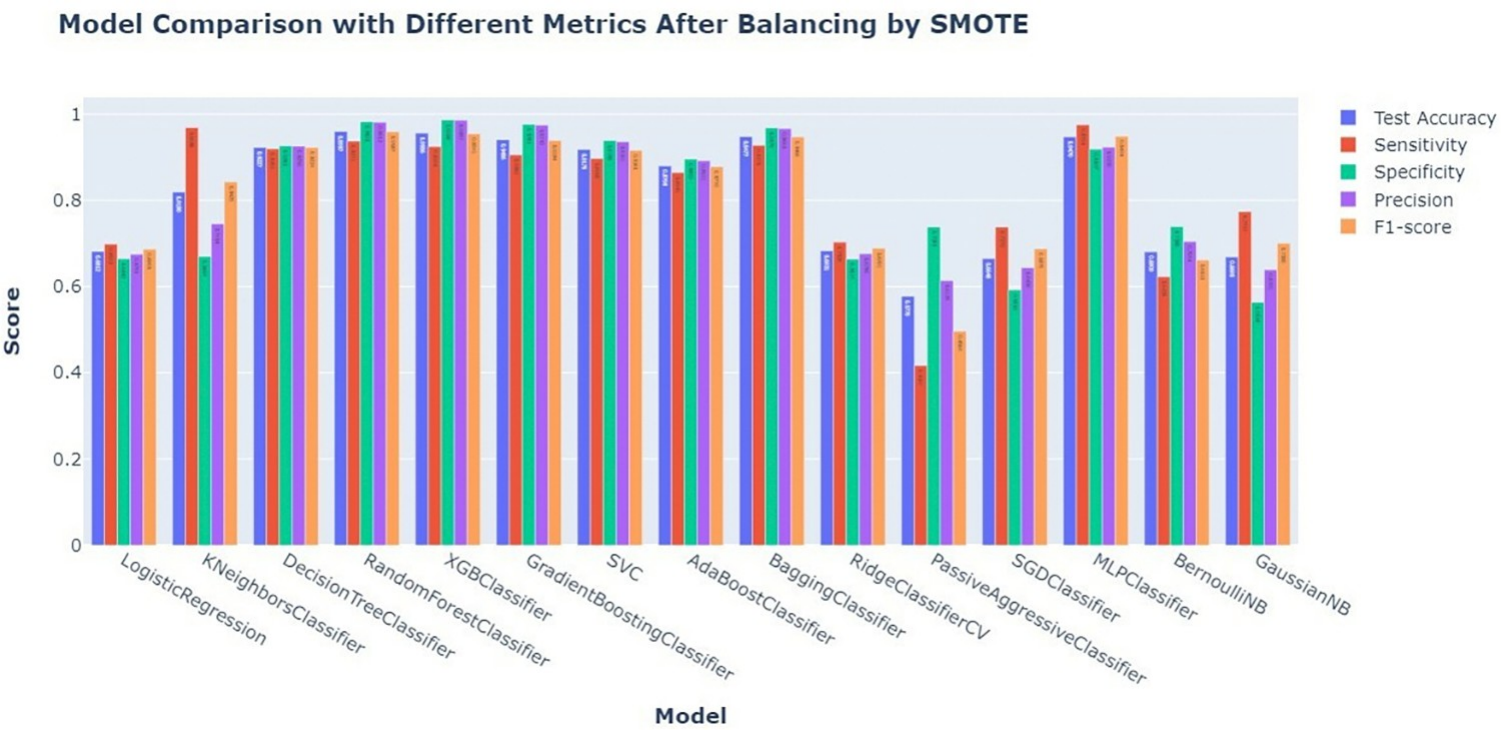
Model comparison with different metrics after balancing by SMOTE.

After balancing the dataset with SMOTE, we thoroughly examined multiple machine learning models based on important performance parameters such as test accuracy, sensitivity, specificity, precision, and F1-score. The results showed that most models performed well, with the Random Forest Classifier, Gradient Boosting Classifier, Bagging Classifier, and XGB Classifier emerging as the best performers. These models scored close to 1.0 on all measures, proving their ability to handle balanced data and make accurate predictions. However, not all models performed equally well on the balanced dataset. Logistic Regression and KNN Classifier performed moderately, with some variation in Sensitivity and F1-score, indicating potential issues in detecting all true positives. PassiveAggressiveClassifier, SGDClassifier, and Naive Bayes variants (BernoulliNB and GaussianNB) showed poorer scores, particularly in Sensitivity and Specificity, indicating that they might not be as effective in distinguishing between the classes even after the application of SMOTE. The use of SMOTE successfully enhanced the performance of most models, particularly those known for their robustness, like ensemble methods (**Fig 7**).

After SMOTE was used to balance the dataset, the ROC curves displayed below showcase the performance of different machine-learning models. The Random Forest Classifier stands out as the best performer with an AUC of 0.99, followed closely by the Bagging Classifier, MLPClassifier, Gradient Boosting Classifier, and XGB Classifier, all of which have AUCs close to or equal to 0.98. These models exhibit nearly error-free ROC curves, indicating their ability to distinguish between classes effectively in the balanced dataset.

**Fig 8:**
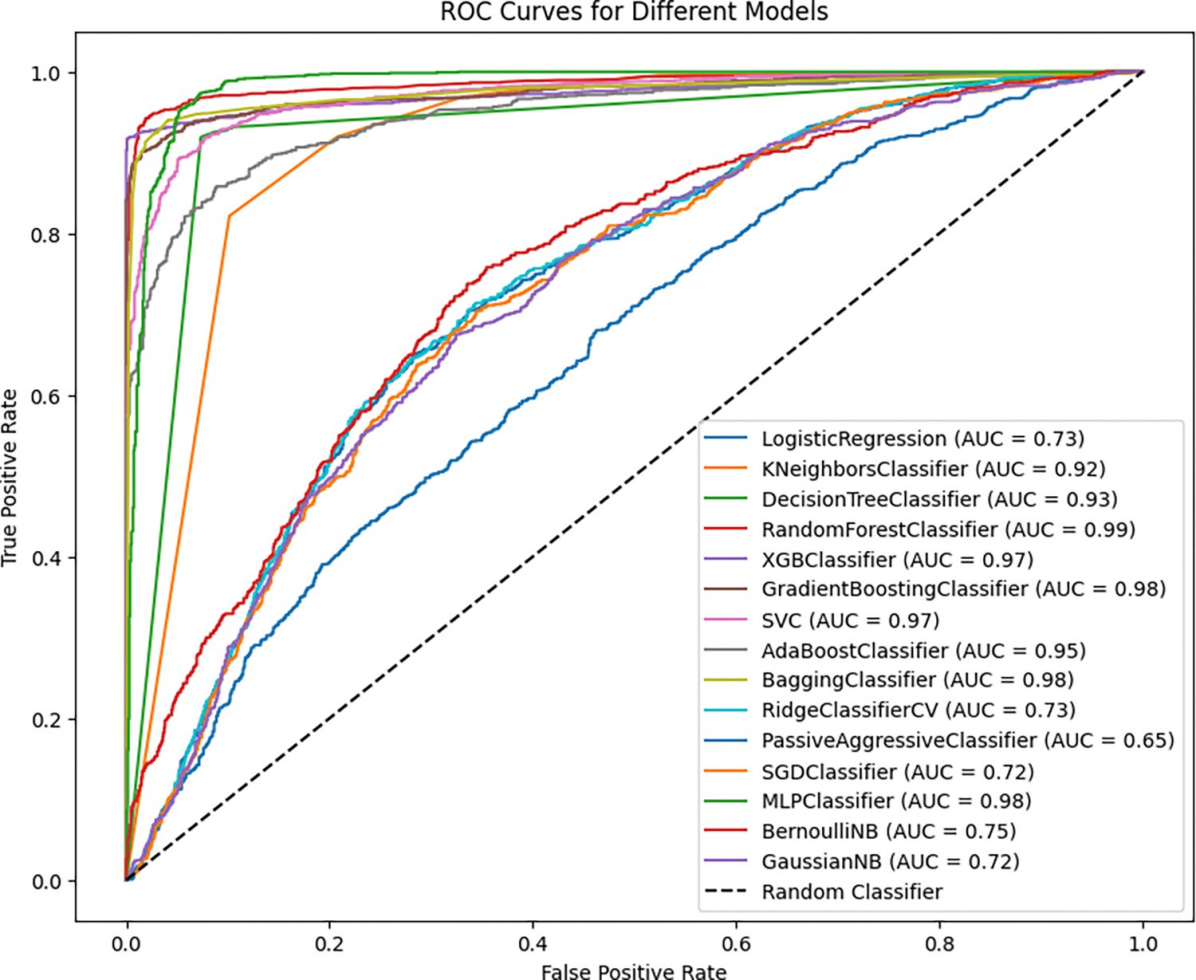
ROC-AUC Comparison after balancing by SMOTE (80%, 20%).

In contrast, the PassiveAggressiveClassifier, SGDClassifier, and GaussianNB models performed poorly, with AUCs of 0.67, 0.73, and 0.72, respectively. These lower AUC values suggest that these models struggle more with the balanced data, possibly indicating that they are less robust in handling the complexity of the dataset (**Fig 8**).

### Experiment 3. Stratified 10-fold Cross-Validation before SMOTE

**Fig 9:**
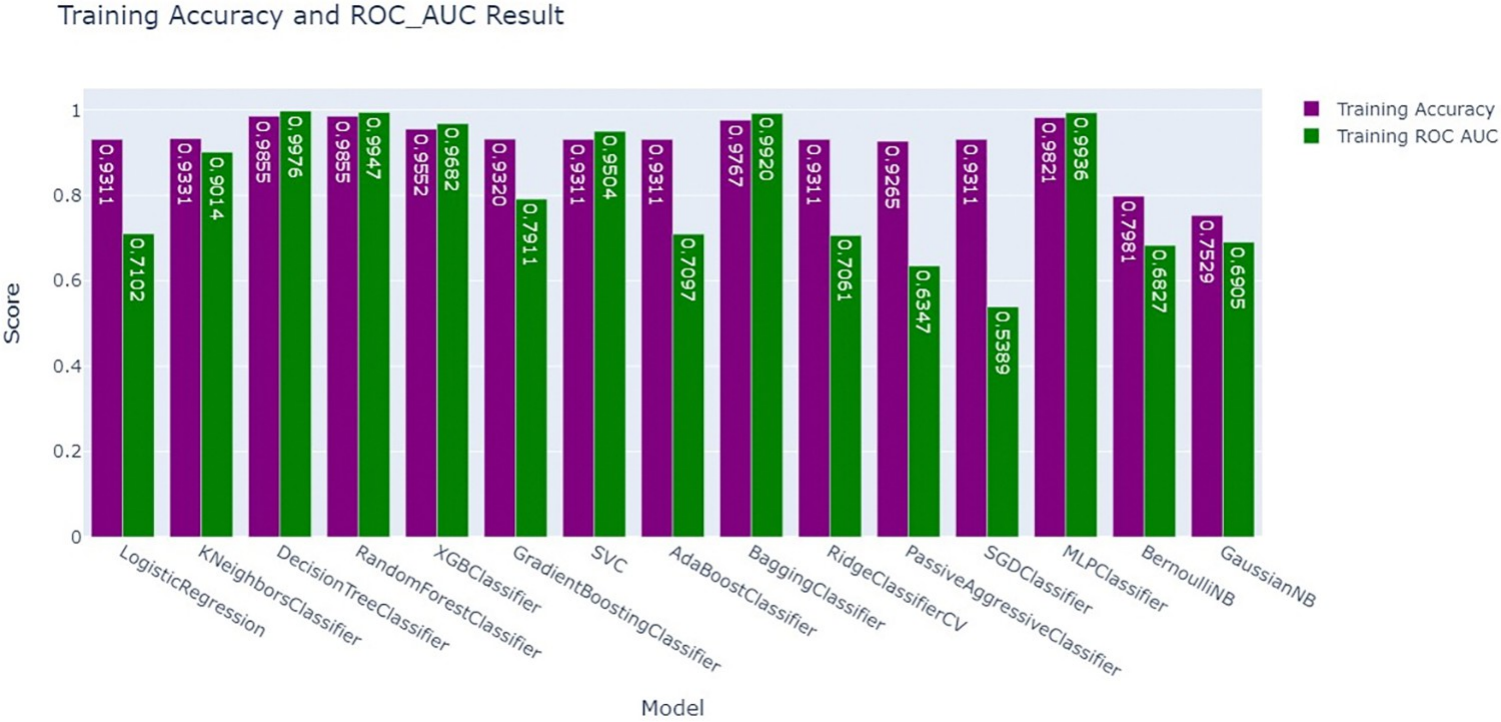
Training Accuracy and ROC-AUC comparison before balancing by SMOTE.

In the stratified 10-fold cross-validation, the Decision Tree Classifier achieved the highest accuracy score of 0.9855 without using SMOTE. This demonstrates its strong ability to detect complex patterns in the data. The Random Forest Classifier followed closely with an accuracy of 0.9853, showcasing its ability to handle a wide range of features while maintaining good performance. The XGB Classifier achieved an accuracy of 0.9509, slightly lower than the top performers, indicating its capacity to model interactions. The MLPClassifier and RidgeClassifierCV also performed well, with accuracies of 0.9714 and 0.9770, showing good generalization throughout the dataset. Meanwhile, the KNN Classifier and the remaining classifier both had comparable accuracy scores of 0.9327, indicating modest performance with potential for improvement through tuning. The GaussianNB classifier achieved the lowest accuracy at 0.7224, suggesting difficulties in adapting to the dataset’s characteristics, potentially because it assumed feature independence (**Fig 9**).

**Fig 10:**
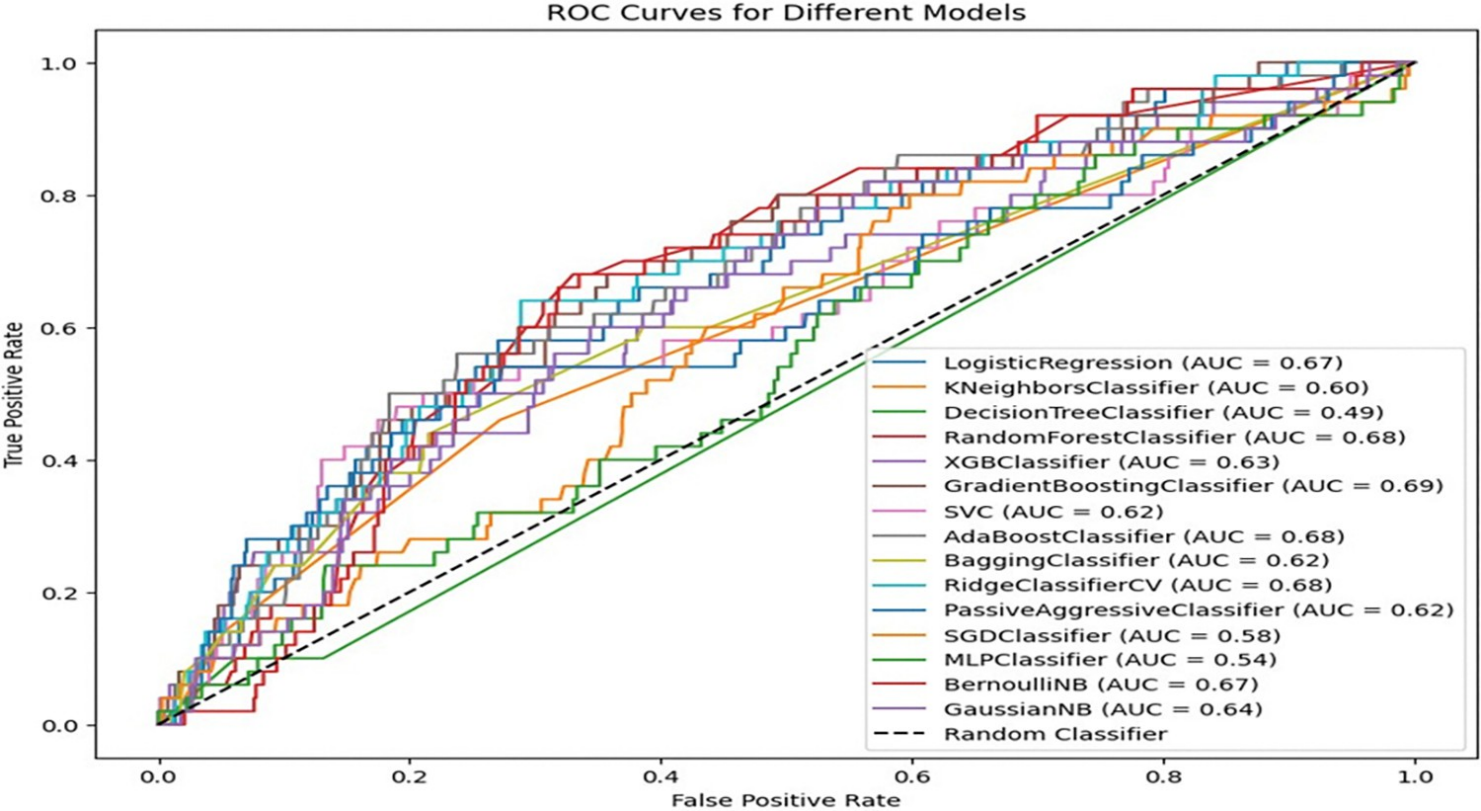
ROC-AUC comparison before balancing by SMOTE (Tenfold CV).

The ROC curves generated using stratified 10-fold cross-validation before SMOTE demonstrate varied levels of model performance on an unbalanced dataset. Models such as Random Forest Classifier, AdaBoost Classifier, RidgeClassifierCV, and Gradient Boosting Classifier have much higher AUC values ranging from 0.68 to 0.69, indicating a moderate capacity to distinguish between classes despite the dataset’s imbalance. In contrast, the Decision Tree Classifier significantly underperforms, with an AUC of 0.49, meaning that its performance is just marginally better than random guessing, most likely due to overfitting to the minority class. Logistic Regression, KNN Classifier, and SVC models perform reasonably well, with AUCs ranging from 0.60 to 0.67. The models tend to be somewhat resistant to class imbalance, while there is still space for development. However, models such as MLPClassifier and SGDClassifier fared badly, with AUCs as low as 0.54 and 0.58, demonstrating difficulty in generalizing well to unbalanced data. As a result, the findings suggest that the imbalanced dataset has a considerable impact on the majority of models, as evidenced by their mediocre to terrible AUC values. These findings emphasize the importance of methods such as SMOTE in balancing the dataset and improving model performance, as underlined in the following analyses(**Fig 10**).

### 4. Stratifying 10-fold Cross-Validation with SMOTE

Stratified 10-fold cross-validation with the SMOTE method provides useful information on the performance of various machine learning models when dealing with imbalanced datasets. Logistic Regression, for example, performs moderately, with an accuracy of 0.6639 and balanced sensitivity and specificity, implying a good capacity to identify PNC utilization despite the initial class imbalance. However, the model’s F1-score of 0.6749 indicates that, while it performs well, there is potential for development, particularly in more complicated cases. The KNN Classifier has a substantially better sensitivity of 0.9746, showing a greater capacity to detect affirmative cases. However, this comes at the cost of specificity, which is significantly lower at 0.6622. This trade-off is shown in the F1-score of 0.8431; this implies that, while the model is successful at detecting PNC use, it may also generate a higher number of false positives, which is problematic in real-world situations where precision is critical.

The Random Forest Classifier and XGB Classifier achieved high accuracies of 0.9552 and 0.9559, respectively, along with strong F1 scores, indicating excellent performance. These models balance sensitivity and specificity well, with the Random Forest Classifier slightly outperforming the others. They demonstrated the effectiveness of ensemble techniques in handling imbalanced data, especially when combined with SMOTE. Conversely, models like the Passive Aggressive Classifier and SGDClassifier performed poorly, with the former exhibiting high sensitivity but low specificity, resulting in an overall accuracy of only 0.5190. The SGDClassifier, with an accuracy of 0.6258, fails to balance sensitivity and specificity, reflecting its difficulties in dealing with imbalanced datasets even after the use of SMOTE **(**Table 4**).**

**Table 4:** Model comparison using multiple metrics after balancing by SMOTE (Tenfold CV).

Model	Test Accuracy	Sensitivity	Specificity	Precision	F1-score
LogisticRegression	0.6639	0.6970	0.6308	0.6541	0.6749
KNeighborsClassifier	0.8185	0.9746	0.6622	0.7429	0.8431
DecisionTreeClassifier	0.9335	0.9254	0.9417	0.9408	0.9330
RandomForestClassifier	0.9552	0.9358	0.9746	0.9736	**0.9543**
XGBClassifier	0.9559	0.9239	0.9880	0.9872	**0.9545**
GradientBoostingClassifier	0.9462	0.9060	0.9865	0.9854	0.9440
SVC	0.9208	0.8985	0.9432	0.9406	0.9191
AdaBoostClassifier	0.8648	0.8507	0.8789	0.8756	0.8630
BaggingClassifier	0.9507	0.9313	0.9701	0.9689	**0.9498**
RidgeClassifierCV	0.6654	0.6970	0.6338	0.6559	0.6758
PassiveAggressiveClassifier	0.5190	0.9701	0.0673	0.5102	0.6687
SGDClassifier	0.6258	0.8478	0.4036	0.5874	0.6940
MLPClassifier	0.9537	0.9776	0.9297	0.9330	**0.9548**
BernoulliNB	0.6714	0.6209	0.7220	0.6910	0.6541
GaussianNB	0.6453	0.7567	0.5336	0.6190	0.6810

**Fig 11:**
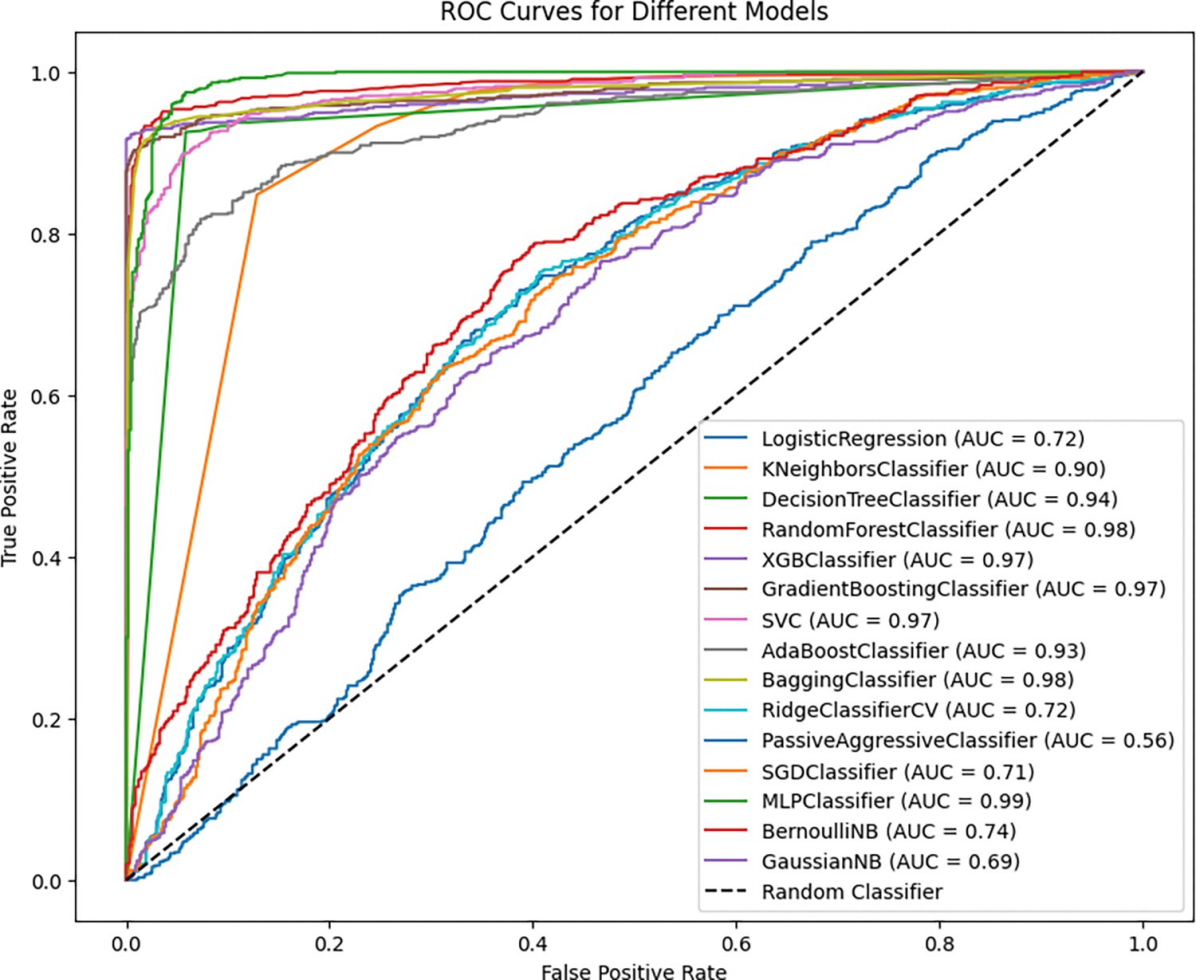
ROC-AUC comparison after balancing by SMOTE (Tenfold CV).

This ROC curve graph compares the performance of various machine learning models in terms of classifying between positive and negative classes. The ROC curve compares the True Positive Rate (sensitivity) to the False Positive Rate (1-specificity) at various classification thresholds, with the area under the curve (AUC) indicating the model’s discriminative capabilities. From fifteen models, the MLP Classifier (AUC = 0.99), Random Forest Classifier (AUC = 0.98), and Bagging Classifier (AUC = 0.98) performed well with a strong capacity for distinguishing between classes. These models have ROC curves that hug the upper left corner of the figure, indicating nearly flawless classification. Compared with the best-performing models, models such as logistic regression (AUC = 0.72), RidgeClassifierCV (AUC = 0.72), and SGDClassifier (AUC = 0.71) have poorer discriminative ability than the best-performing models. The ROC curves are more centered and near the diagonal line, which indicates random guessing. The PassiveAggressiveClassifier has the lowest AUC of 0.56, showing poor class differentiation. Its ROC curve is the closest to the diagonal, indicating that its classification capability is marginally superior to random guessing (**Fig 11**).

### Feature selection

**Fig 12:**
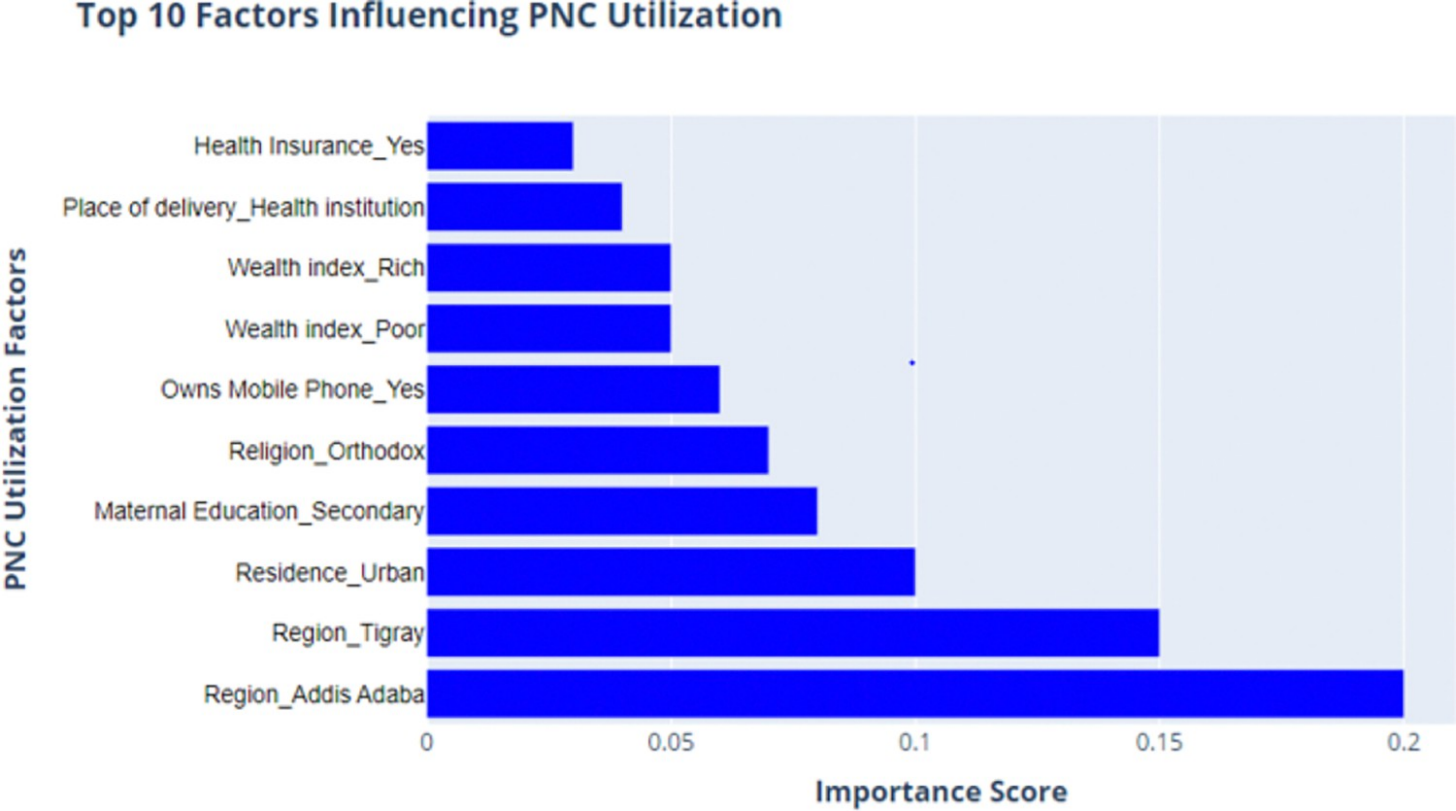
Top 10 features influencing PNC utilization.

The bar chart illustrates the top 10 factors that influence postnatal care (PNC) utilization, ranked by their importance scores in predicting PNC utilization. The most influential factor is the region, particularly Region_Addis Ababa, with an importance score of nearly 0.2, indicating a significant impact of geographical location, specifically from Addis Ababa, on PNC service utilization. The second most important factor is Region_Tigray, followed by Residence_Urban, highlighting the critical role of urban residency and regional differences in PNC utilization. Other factors such as maternal education, religion, and wealth index also contribute significantly, though to a lesser extent. For example, Maternal Education_Secondary and Religion_Orthodox have moderate importance scores, suggesting that educational background and religious affiliation influence PNC utilization decisions. Additionally, factors like health insurance status and the place of delivery, such as delivering in a health institution, though less influential, still contribute meaningfully to predicting PNC utilization (Fig 12)

## Discussion

Among the four experimental designs, tenfold cross-validation with SMOTE balancing generated the best performance. This study focused on predicting postnatal care utilization using machine learning, a topic with significant implications for the health outcomes of mothers and children. Using various machine learning models, the analysis of postnatal care utilization data reveals important insights into the factors influencing PNC utilization and the effectiveness of different predictive models. This study employed four distinct experimental setups to evaluate 14 machine learning algorithms, comparing their performance with and without the implementation of the Synthetic Minority Over-sampling Technique (SMOTE) to address the prevalent issue of class imbalance within the dataset [72]. This comprehensive approach enables a thorough assessment of each model’s capacity to generalize to new data, which is crucial for developing reliable predictive models for real-world applications [73,74].

The first experiment used a standard 80%-20% train-test split without SMOTE. A notable result occurred. Models like the Decision Tree Classifier (98.59%), Random Forest Classifier (98.57%), MLP Classifier (98.24%), Bagging Classifier (97.50%), and XGBoost Classifier (95.69) had great training accuracy but low sensitivity. This disparity, which is frequent in unbalanced datasets, suggests that these models are prone to overfitting the majority class, hindering the discovery of actual positive PNC utilization cases [75] [39]. These models have a significant ability to reliably detect unutilized PNC, but they struggle to accurately forecast actual PNC utilization, which is a critical factor in healthcare applications [76,77]. These findings highlight the importance of considering evaluation criteria beyond accuracy, especially when working with imbalanced datasets. The sensitivity, F1-score, and ROC AUC are more comprehensive and insightful measures of a model’s performance. The observed ROC AUC scores ranged from 0.77 for Logistic Regression to 0.50 for some classifiers, emphasizing the importance of using approaches such as SMOTE to address the class imbalance [78].

When SMOTE was added to the 80%-20% train-test split experimental design, the performance of most models improved significantly, particularly for ensemble approaches like Random Forest, Gradient Boosting, and Bagging. These models are well-known for their robustness, as evidenced by consistently high results on all tested criteria such as sensitivity, specificity, accuracy, and F1 score. This shows that SMOTE effectively lowers class imbalances while also improving the models’ ability to learn from minority class occurrences [79,80]. This enhancement leads to more accurate and trustworthy predictions, which are critical for healthcare applications in detecting people in need of PNC. Even when was used SMOTE, many models, such as Logistic Regression and the k-Nearest Neighbors classifier, revealed minimal improvements. These findings indicate that these models may need to be refined further or alternate approaches investigated to produce equivalent findings [81]. A closer look at the ROC curves reveals solid evidence that SMOTE improves model performance. After using SMOTE, models such as Random Forest(AUC = 0.99), Bagging(AUC = 0.98), and MLP Classifier(AUC = 0.98) showed near-perfect ROC curves with AUC scores close to 1.0, indicating their ability to distinguish between classes within the balanced dataset[82]. Even after dataset balancing, models such as Passive Aggressive Classifier and SGDClassifier have much lower AUC values. This mismatch reveals critical limits in these models, implying difficulty in successfully handling the complexities inherent in the data, even when class imbalance is handled [83].

In the third and fourth experiments, moving from a single train-test split to stratified 10-fold cross-validation allowed for a more comprehensive assessment of the model’s generalization capacity. This method provides a more trustworthy assessment by replicating multiple train-test splits, especially those with minimal data, thus reducing the degree of uncertainty associated with a single split [84]. These findings highlight the need to resolve class imbalance to increase the reliability of model performance evaluation both before and after SMOTE implementation [41]. Tenfold cross-validation with balancing using Synthetic Minority Over-sampling Technique was outperformed. From fifteen models, the MLP Classifier (f1-score = 0.9548, AUC = 0.99), Random Forest Classifier (f1-score = 0.9543, AUC = 0.98), and Bagging Classifier (f1-score = 0.9498, AUC = 0.98) performed excellently, with a strong ability to differentiate between classes. The study’s findings highlight an essential element of machine learning: choosing between train-test split and cross-validation does not imply that one strategy is necessarily better than the other. Instead, it emphasizes the importance of knowing the strengths and limits of each technique related to the particular study topic and data features. Cross-validation enables a more comprehensive and reliable evaluation, particularly when dealing with limited or imbalanced data, which is prevalent in healthcare research. A simple train-test split could be enough for preliminary investigations, particularly with large amounts of data [85].

This study not only analyzed model performance but also aimed to identify the key factors influencing PNC utilization. Region, residence, maternal education, religion, wealth index, health insurance status, and place of delivery are identified as contributing factors that predict postnatal care utilization. These findings are consistent with previous research in India [86], Nepal [87], Nigeria [88], and Ethiopia [89–92]. Region was a significant predictor of postnatal care utilization. It was found that geographic location, particularly living in the Addis Ababa and Tigray regions significantly increased the likelihood of using PNC services. This finding aligns with the literature that highlights the influence of geographical accessibility and regional disparities on healthcare utilization [26,89].

The findings of this study have significant policy and practice implications for mothers’ and children’s health. The observation of geographical differences in PNC utilization emphasizes the need for targeted efforts to improve access to services in marginalized areas. This could include improving healthcare infrastructure in rural places, deploying mobile health clinics to serve underprivileged people, or creating community-based health programs that empower local women to offer basic PNC services. Addressing the impact of socioeconomic variables necessitates a cross-sectoral approach that addresses issues such as poverty, a lack of education, and gender inequality. This could include activities such as conditional cash transfer programs that encourage PNC utilization, improve girls’ education to empower women to make informed health decisions, and address cultural issues. However, it is crucial to acknowledge the limitations of this study. Compared with statistical models, machine learning models lack interpretability as they do not provide coefficients or odds ratios, making it difficult to assess the impact of various factors. To address this limitation, the researchers conducted additional analyses to determine how factors influence PNC utilization. The second limitation of this study is its reliance on the 2016 EDHS datasets, which are outdated compared to more recent datasets. This choice was necessitated by the lack of recent national surveys. To effectively compare and evaluate the performance of the models in predicting PNC utilization, the study required a substantial amount of data, resulting in the use of the 2016 EDHS datasets. The third limitation of this study is the lack of external validation, which restricts the generalizability of our findings beyond the Ethiopian Demographic and Health Survey (EDHS) data. Without testing the model on independent datasets, we cannot ascertain its performance in diverse contexts or populations. To address this limitation, future research should prioritize the application of the model to external datasets.

## Conclusion

This research paper evaluates four experimental designs for predicting postnatal care (PNC) utilization, concluding that tenfold cross-validation combined with SMOTE is the most effective approach. Among the machine learning models tested MLP Classifier, Random Forest Classifier, and Bagging Classifier each demonstrated strong predictive capabilities, highlighting their reliability in healthcare applications. Key findings indicate that PNC utilization is significantly influenced by factors such as region, maternal education, religion, wealth index, health insurance status, and place of delivery. This underscores the importance of regional and socioeconomic determinants in accessing maternal healthcare services. The study emphasizes the effectiveness of machine learning in tackling healthcare challenges and the need to address class imbalances in healthcare data to enhance model performance and ensure equitable health outcomes. By understanding these influencing factors, the research aims to inform targeted interventions and policies, contributing to improving maternal and child health and promoting health equity in care access.
